# Hydrolethalus Syndrome: A Case of a Rare Congenital Disorder

**DOI:** 10.3390/diagnostics15020202

**Published:** 2025-01-17

**Authors:** Valerica Belengeanu, Diana Marian, Horia Ademir Stana, Carolina Cojocariu, Cristina Popescu, Ioana Elena Lile

**Affiliations:** 1 Department of Medicine, Faculty of Medicine, “Vasile Goldiș” Western University of Arad, 94-96 Revolutiei Blvd., 310025 Arad, Romania; belvtim@yahoo.com (V.B.); stana.ademirhoria@yahoo.com (H.A.S.); 2 Department of Dentistry, Faculty of Dentistry, “Vasile Goldiș” Western University of Arad, 94-96 Revolutiei Blvd., 310025 Arad, Romania; carolina.cojo@yahoo.com (C.C.); lile.ioana@uvvg.ro (I.E.L.); 3 Faculty of Pharmacy, “Vasile Goldiș” Western University of Arad, 94-96 Revolutiei Blvd., 310025 Arad, Romania; pursega36@gmail.com

**Keywords:** hydrolethalus syndrome, polyhydramnios, palate cleft, labial cleft, micrognathia, skeletal anomalies

## Abstract

This is a fatal case of multiple complicated congenital anomalies displaying several symptoms consistent with hydrolethalus syndrome. The newborn’s phenotype is characterized by a combination of serious anatomical abnormalities such as open-book cerebral hemispheres, defective lobulation of the lungs (one lobe on the left, two on the right), a smaller right kidney, a smooth cerebral surface, and a specific keyhole-shaped defect in the skull base, primarily associated with hydrocephalus.

**Figure 1:**
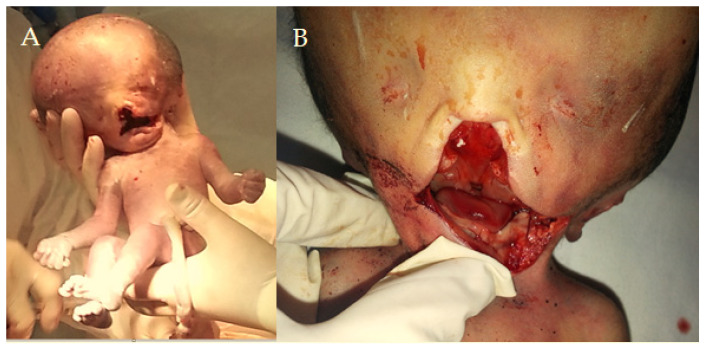

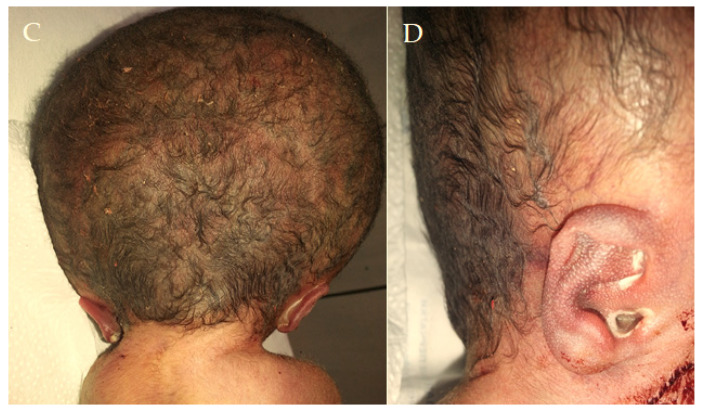
This newborn’s phenotype is mainly associated with hydrocephalus, along with other anatomical anomalies such as open-book cerebral hemispheres, defective lobulation of the lungs (one lobe on the left and two lobes on the right), a smaller right kidney, a smooth cerebral surface, and a specific keyhole-shaped defect in the skull base, as also described by Pateau [1]. Hydrolethalus syndrome (HLS) is a rare and severe genetic disorder with an autosomal recessive inheritance pattern, marked by neurological and physical problems that primarily affect the brain and midline structures. The primary cause is represented by mutations in the HYLS1 gene on chromosome 11q24 [1]. This gene encodes a protein that plays a crucial role in centrosome and ciliary functions, vital for accurate cell division and signaling during embryonic development. The term hydrolethalus comprises the key features of the syndrome: polyhydramnios, hydrocephalus, and the potential for a high lethality rate, with the majority of affected infants either stillborn or succumbing shortly after birth. Notable characteristics include hydrocephalus, micrognathia, polydactyly, and distinct skeletal anomalies [1]. Understanding these key features is essential for better diagnosis and intervention. Salonen et al. coined the term “hydrolethalus syndrome” in 1981 to describe patients with severe central nervous system malformations [2]. There is a diverse group of genetic disorders in which abnormalities of nervous system development associated with cleft lip/palate and polydactyly, such as the 14 different types of OFD syndrome, are predominant [3]. The physical examination after birth revealed a complex condition (**A**), with disproportionately large hypertelorism with underdeveloped orbits, anophthalmia, a broad anomalous nose, and a significant median cleft in the upper jaw impacting the base of the nose, the palate, and the upper lip (**B**), along with a short lower jaw and small ears positioned very low (**C,D**). After 29 unfollowed weeks of gestation, the infant was delivered via an urgent cesarean section, presenting multiple anomalies. The Roma parents, who have not declared a consanguineous marriage (the mother is 17 years old, and the father is 20 years old), also have a 2-year-old child with ventricular septal defect (VSD). Before the caesarian, an ultrasound investigation was performed. The mother showed signs of polyhydramnios, whereas the fetus, weighing 1690 g, had an FL (femur length) of 5 cm and a cranial perimeter of 40.61 cm. The fetus also had massive hydrocephalus and corpus callosum agenesis. The first description of HLS dates from the early 1980s in Finland, where the incidence is 1:20,000 [4]. Few cases have been recorded elsewhere, and the pathophysiology is unknown. In Finland, researchers identified a founder missense variant (c.632A > G, p. [Asp211Gly]) in the HYLS1 gene (11q24.2). This pathogenic variant is sporadic outside the Finnish population. Patients with HILS2-like phenotypes can also have mutations in the KIF7 gene (15q26.1) [5]. Putoux et al. describe a consanguineous Algerian family in which four sib fetuses had a lethal developmental disorder consistent with hydrocephalus syndrome [5]. In these cases, some patients had anencephaly, and others had hydrocephalus. The KIF7 mutation has been described in only one HLS family. Both HYLS1 and KIF7 genes encode ciliary or centriolar proteins that appear to be involved in early embryonic midline development. The typical thumb duplication is a characteristic of the clinical picture in HILS1 syndrome, but these findings are not present in this case. The range for HYLS1 and the severity of physical characteristics may vary from case to case [5]. The involvement of multiple structures in this case is particularly remarkable, with this being the particularity of the hydrolethalus syndrome.

**Figure 2:**
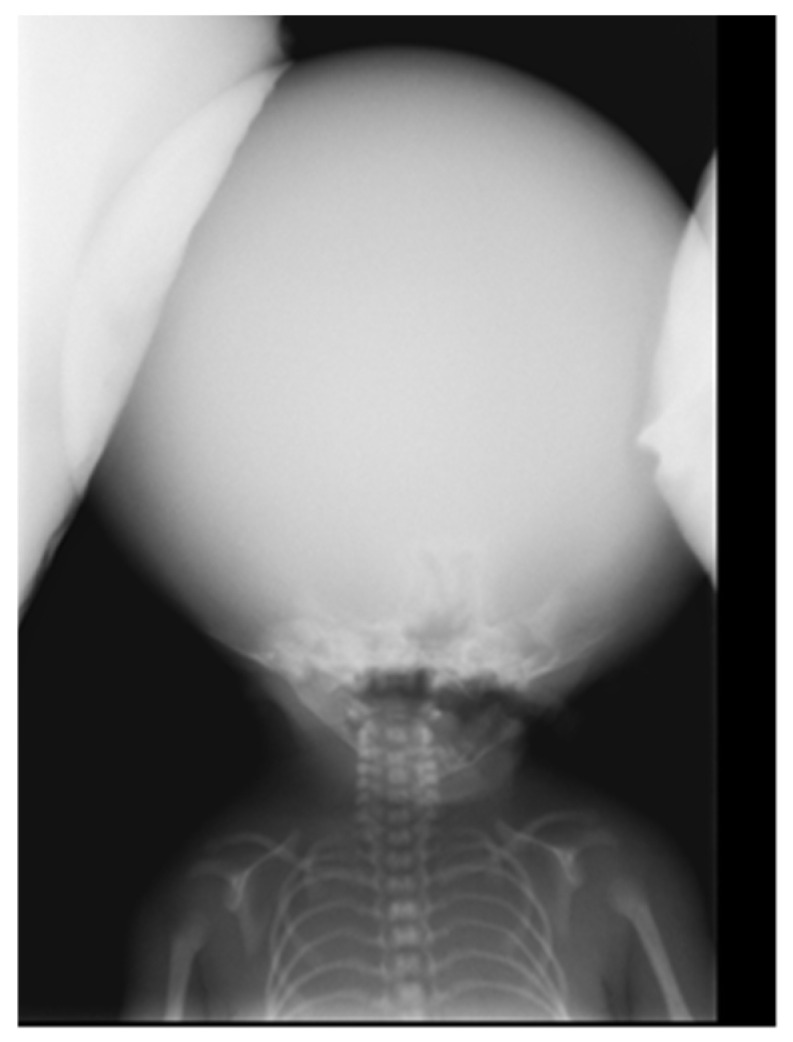
The X-ray image illustrates a significant skull expansion, confirming hydrocephalus, comprising macrocephaly, characterized by a disproportionately large head because of excessive cerebrospinal fluid accumulation inside the cranial cavity, along with a reduced bone density of the skull.

**Figure 3:**
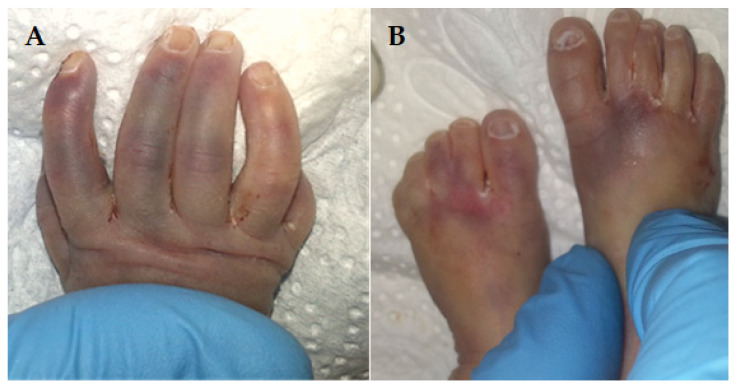
Other anomalies include right-hand polydactyly, central for the III-rd finger (**A**), severe bilateral clinodactyly in finger V (**A**), and bilateral simian creases. Additionally, there is bilateral syndactyly affecting the legs in fingers II/III (**B**), an absence of mammary areolas, and an absence of the small labia.

**Figure 4:**
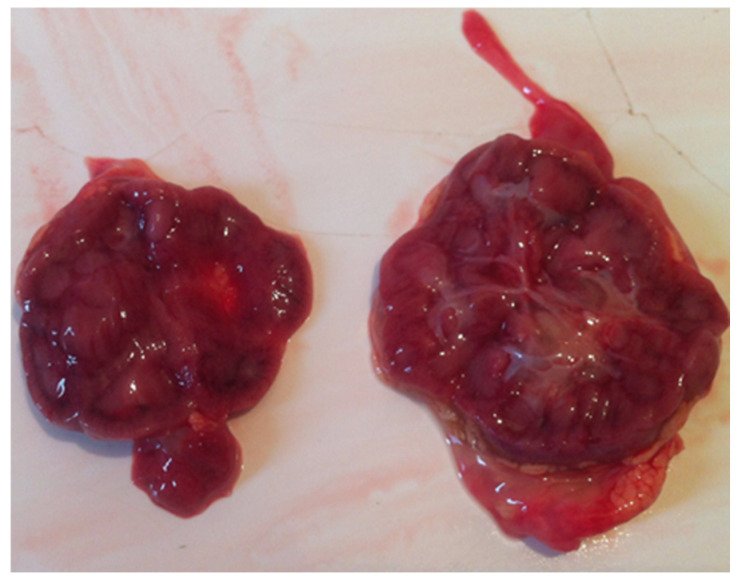
Image of the kidneys. The right kidney is smaller than the left one. Other anomalies include ventricular septal defect (VSD), defective lobulation of the lungs, a right lung with two lobes instead of three, and a left lung with only one lobe. The right kidney is tiny compared to the left.

**Figure 5:**
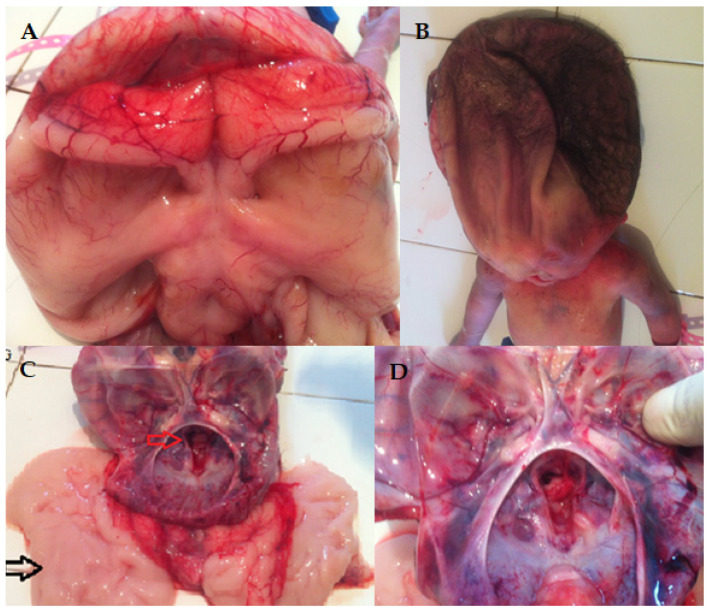
The newborn lived for 10 min. The autopsy findings of the brain revealed “open-book” cerebral hemispheres (**A**), a skull cap of soft consistency (**B**), a smooth cerebral surface (black arrow) (**C**), and a keyhole-shaped defect in the occipital bone (red arrow) (**C**,**D**) as an abnormally widened aperture, resembling the shape of a keyhole. This often occurs near the foramen magnum or adjacent cranial base structures [1]. The keyhole shape may result from insufficient ossification of the surrounding skull bones. It is a distinctive morphological clue in diagnosing hydrocephalus syndrome, helping to distinguish it from other disorders with similar features [1]. The phenotypic manifestations, along with those found on autopsy, support the diagnosis of hydrolethalus syndrome (HLS). The triad of polyhydramnios, hydrocephalus, and lethality is characteristic of HLS, which belongs to the ciliopathies group. HLS is typically characterized by severe central nervous system malformations, with hydrocephalus and absent midline structures. Exome sequencing of *HYLS1* and *KIF7* genes did not identify a disease-causing variant, either in the proband or the parents. Sanger sequencing of *HYLS1* chr11:125761319-125761367 bp, hg19 region, whose variant could have been responsible for the proband phenotype, was repeatedly unsuccessful, most likely due to the size or quality of fragments of PCR (Polymerase Chain Reaction) products. The inability to detect pathogenic variants presents significant limitations in the context of genetic analysis, particularly when diagnosing conditions with complex or rare genetic etiologies. These limitations arise from both technical and biological factors. Pathogenic variants of technically challenging types for NGS (Next-Generation Sequencing) require advanced bioinformatics tools and a combination of sequencing methods in order to detect and characterize them accurately. These heterogeneous challenging types include large indels, small CNVs (Copy Number Variations), mobile element insertions, complex rearrangements, and variants within segmental duplications or low-complexity regions. No current NGS platform or variant calling algorithm can capture all these variant types. All high-throughput sequencing options generate large volumes of raw data that make pathogenic variant detection and identification challenging. An interlaboratory study shows that missed variants were visible in most raw data sets, indicating that the sensitivity limitations were essentially bioinformatics [6]. On the other side, whole exome sequencing (WES) has some technical limitations, such as the incomplete coverage of exonic regions, lack of coverage of deep intronic and regulatory regions, the introduction of PCR artifacts during library preparations, and uneven sequence depth [7,8,9]. Since the WES and Sanger analysis were externalized to an accredited lab, we believe that the inability to detect pathogenic variants may be attributed to either library preparation or the laboratory algorithm. Unfortunately, the quantity of biological material obtained from the proband was insufficient to proceed with WGS (whole genome sequencing) or CMA (Chromosomal Microarray Analysis). Nonetheless, whole exome sequencing (WES) has the potential to identify novel genes involved in ciliopathy pathogenesis, while Comparative Genomic Hybridization (CGH) remains a reliable clinical tool for detecting microdeletions or microduplications, offering complementary insights into complex genetic disorders. Also, for differential diagnosis, Fryns syndrome was considered due to the cleft lip and palate, polyhydramnios, and mortality. However, we could rule out this condition because polydactyly was present, and distal digital hypoplasia, a coarse face, and diaphragm abnormalities were absent [10]. Also, the diagnosis of Meckel syndrome was excluded in the absence of characteristic features such as anencephaly, as well as kidney and hepatic dysgenesis. Hydrocephaly is a rare manifestation associated with Meckel syndrome. However, it may occur [11]. In acrocallosal syndrome, macrocephaly is present without hydrocephaly, hypertelorism, polysyndactyly of the hands/feet, or a cleft lip/palate, features that were present in this case; therefore, the acrocallosal syndrome was excluded [12]. Pallister–Hall syndrome also has standard features with hydrolethalus, including micrognathia, polydactyly, defective lobulation of the lungs, congenital heart defects, and occasionally a cleft lip and/or palate but is not associated with open-book brain abnormality [13]. Hydrolethalus syndrome (HLS) is a rare genetic disorder, with only a limited number of cases recorded globally. The infrequency of HLS poses difficulties in accurately estimating its global incidence; nonetheless, it is regarded as relatively low, with only a limited number of cases documented in the medical literature. The significance of isolated cases of hydrolethalus syndrome cannot be overstated. Every new case offers essential insights into the fundamental genetic and molecular mechanisms that play a role in the development of the syndrome. These uncommon instances contribute to clarifying the phenotypic range of HLS, enhancing diagnostic standards, and assisting in genetic counseling for impacted families. More importantly, isolated cases enable researchers to investigate possible connections to other comparable disorders, discover new pathogenic variants, and ultimately aid the progress of precision medicine within ciliopathies and associated genetic conditions. Due to the infrequency of HLS, researchers and clinicians must continue to document and publish comprehensive case reports. This joint initiative will deepen our comprehension of the syndrome’s clinical features and facilitate new research opportunities. This case contributes significantly to our understanding of hydrolethalus syndrome (HLS) by expanding the phenotypic spectrum of the disorder and unraveling its complex pathogenesis. The unique presentation of this patient, characterized by a keyhole-shaped defect in the skull, open-book cerebral hemispheres, abnormal lobulation of the lungs, and a range of other anatomical anomalies, serves as a powerful reminder of the diverse nature of HLS. It also underscores the crucial role of meticulous clinical documentation in rare genetic disorders. These findings confirm previously reported features and offer fresh insights into the variability of the syndrome, which can be instrumental in refining diagnostic criteria. Also, this case underscores the importance of integrating clinical, imaging, and pathological observations to understand HLS thoroughly. In similar situations, the absence of molecular confirmation further underscores the need for advanced genetic testing techniques, such as whole exome sequencing (WES) or whole genome sequencing (WGS). These techniques can potentially uncover novel variants or genes linked to the disorder, thereby enhancing our understanding of HLS.

## References

[1] Paetau A., Honkala H., Salonen R., Ignatius J., Kestilä M., Herva R. (2008). Hydrolethalus syndrome: Neuropathology of 21 cases confirmed by HYLS1 gene mutation analysis. J. Neuropathol. Exp. Neurol..

[2] Salonen R., Herva R. (1990). Hydrolethalus syndrome. J. Med. Genet..

[3] Belengeanu V., Marian D., Hosszu T., Ogodescu A.S., Belengeanu A.D., Samoilă C., Freiman P., Lile I.E. (2019). A comprehensive evaluation of an OFDI syndrome from child to teenager. Rom. J. Morphol. Embryol..

[4] Visapää I., Salonen R., Varilo T., Paavola P., Peltonen L. (1999). Assignment of the locus for hydrolethalus syndrome to a highly restricted region on 11q23-25. Am. J. Hum. Genet..

[5] Putoux A., Thomas S., Coene K.L., Davis E.E., Alanay Y., Ogur G., Uz E., Buzas D., Gomes C., Patrier S. (2011). KIF7 mutations cause fetal hydrolethalus and acrocallosal syndromes. Nat. Genet..

[6] Lincoln S.E., Hambuch T., Zook J.M., Bristow S.L., Hatchell K., Truty R., Kennemer M., Shirts B.H., Fellowes A., Chowdhury S. (2021). One in seven pathogenic variants can be challenging to detect by NGS: An analysis of 450,000 patients with implications for clinical sensitivity and genetic test implementation. Genet. Med..

[7] Meienberg J., Bruggmann R., Oexle K., Matyas G. (2016). Clinical sequencing: Is WGS the better WES?. Hum. Genet..

[8] Lelieveld S.H., Spielmann M., Mundlos S., Veltman J.A., Gilissen C. (2015). Comparison of exome and genome sequencing technologies for the complete capture of protein-coding regions. Hum. Mutat..

[9] Belkadi A., Bolze A., Itan Y., Cobat A., Vincent Q.B., Antipenko A., Shang L., Boisson B., Casanova J.L., Abel L. (2015). Whole-genome sequencing is more powerful than whole- exome sequencing for detecting exome variants. Proc. Natl. Acad. Sci. USA.

[10] Slavotinek A.M. (2004). Fryns syndrome: A review of the phenotype and diagnostic guidelines. Am. J. Med. Genet. A.

[11] Khurana S., Saini V., Wadhwa V., Kaur H. (2017). Meckel-Gruber syndrome: Ultrasonographic and fetal autopsy correlation. J. Ultrasound.

[12] Koenig R., Bach A., Woelki U., Grzeschik K.-H., Fuchs S. (2002). Spectrum of the acrocallosal syndrome. Am. J. Med. Genet..

[13] Hall J.G., Pallister P.D., Clarren S.K., Beckwith J.B., Wiglesworth F.W., Fraser F.C., Cho S., Benke P.J., Reed S.D. (1980). Congenital hypothalamic hamartoblastoma, hypopituitarism, imperforate anus, and postaxial polydactyly. A new syndrome? Part I: Clinical, causal and pathogenetic considerations. Am. J. Med. Genet..

